# Associated factors of poor treatment outcomes in patients with giant cell arteritis: clinical implication of large vessel lesions

**DOI:** 10.1186/s13075-020-02171-6

**Published:** 2020-04-07

**Authors:** Takahiko Sugihara, Hitoshi Hasegawa, Haruhito A. Uchida, Hajime Yoshifuji, Yoshiko Watanabe, Eisuke Amiya, Yasuhiro Maejima, Masanori Konishi, Yohko Murakawa, Noriyoshi Ogawa, Shunsuke Furuta, Yasuhiro Katsumata, Yoshinori Komagata, Taio Naniwa, Takahiro Okazaki, Yoshiya Tanaka, Tsutomu Takeuchi, Yoshikazu Nakaoka, Yoshihiro Arimura, Masayoshi Harigai, Mitsuaki Isobe, Japan Research Committee of the Ministry of Health, Labour, and Welfare for Intractable Vasculitis (JPVAS), Shigeto Kobayashi, Tetsuya Horita, Hiroaki Dobashi, Eri Muso, Atsushi Komatsuda, Satoshi Ito, Kazuo Tanemoto, Hiroshi Akazawa, Issei Komuro, Koichi Amano, Atsushi Kawakami, Takashi Wada, Taichi Hayashi, Shinichi Takeda

**Affiliations:** 1 Department of Lifetime Clinical Immunology, Graduate School of Medical and Dental Sciences, Tokyo Medical and Dental University, 1-5-45 Yushima, Bunkyo-ku, Tokyo 113-8519 Japan; 2 Department of Rheumatology, Graduate School of Medical and Dental Sciences, Tokyo Medical and Dental University, Tokyo, Japan; 3 Department of Medicine and Rheumatology, Tokyo Metropolitan Geriatric Hospital, Tokyo, Japan; 4 Department of Hematology, Clinical Immunology and Infectious Diseases, Ehime University Graduate School of Medicine, Matsuyama, Ehime Japan; 5 Department of Chronic Kidney Disease and Cardiovascular Disease, Okayama University Graduate School of Medicine, Dentistry and Pharmaceutical Sciences, Okayama, Japan; 6 Department of Rheumatology and Clinical Immunology, Graduate School of Medicine, Kyoto University, Kyoto, Japan; 7 First Department of Physiology, Kawasaki Medical School, Kurashiki, Japan; 8 Department of Cardiovascular Medicine, Graduate School of Medicine, Department of Therapeutic Strategy for Heart Failure, The University of Tokyo, Tokyo, Japan; 9 Department of Cardiovascular Medicine, Tokyo Medical and Dental University, Tokyo, Japan; 10 Department of Rheumatology, Shimane University Faculty of Medicine, Izumo, Japan; 11 Department of Internal Medicine 3, Hamamatsu University School of Medicine, Hamamatsu, Japan; 12 Department of Allergy and Clinical Immunology, Chiba University Hospital, Chiba, Japan; 13 Department of Rheumatology, Tokyo Women’s Medical University School of Medicine, Tokyo, Japan; 14 Department of Nephrology and Rheumatology, Kyorin University School of Medicine, Tokyo, Japan; 15 Division of Rheumatology, Department of Internal Medicine, Nagoya City University Hospital, Nagoya, Japan; 16 Department of Respiratory Medicine, Allergy and Clinical Immunology, Nagoya City University Graduate School of Medical Sciences, Nagoya, Japan; 17 Division of Rheumatology & Allergology, Department of Internal Medicine, St. Marianna University School of Medicine, Kawasaki, Japan; 18 National Hospital Organization, Shizuoka Medical Center, Shimizu, Japan; 19 The First Department of Internal Medicine, University of Occupational and Environmental Health, Japan, Kitakyushu, Japan; 20 Division of Rheumatology, Department of Internal Medicine, Keio University School of Medicine, Tokyo, Japan; 21 Department of Cardiovascular Medicine, Osaka University Graduate School of Medicine, Suita, Japan; 22 Department of Vascular Physiology, National Cerebral and Cardiovascular Center Research Institute, Suita, Japan; 23 Kichijoji Asahi Hospital, Tokyo, Japan; 24 Sakakibara Heart Institute, Tokyo, Japan

**Keywords:** Giant cell arteritis, Large vessel lesions, Poor treatment outcomes, Remission, Relapse, Glucocorticoid therapy

## Abstract

**Background:**

Relapses frequently occur in giant cell arteritis (GCA), and long-term glucocorticoid therapy is required. The identification of associated factors with poor treatment outcomes is important to decide the treatment algorithm of GCA.

**Methods:**

We enrolled 139 newly diagnosed GCA patients treated with glucocorticoids between 2007 and 2014 in a retrospective, multi-center registry. Patients were diagnosed with temporal artery biopsy, 1990 American College of Rheumatology classification criteria, or large vessel lesions (LVLs) detected by imaging based on the modified classification criteria. Poor treatment outcomes (non-achievement of clinical remission by week 24 or relapse during 52 weeks) were evaluated. Clinical remission was defined as the absence of clinical signs and symptoms in cranial and large vessel areas, polymyalgia rheumatica (PMR), and elevation of C-reactive protein (CRP) levels. A patient was determined to have a relapse if he/she had either one of the signs and symptoms that newly appeared or worsened after achieving clinical remission. Re-elevation of CRP without clinical manifestations was considered as a relapse if other causes such as infection were excluded and the treatment was intensified. Associated factors with poor treatment outcomes were analyzed by using the Cox proportional hazard model.

**Results:**

Cranial lesions, PMR, and LVLs were detected in 77.7%, 41.7%, and 52.5% of the enrolled patients, respectively. Treatment outcomes were evaluated in 119 newly diagnosed patients who were observed for 24 weeks or longer. The mean initial dose of prednisolone was 0.76 mg/kg/day, and 29.4% received any concomitant immunosuppressive drugs at baseline. Overall, 41 (34.5%) of the 119 patients had poor treatment outcomes; 13 did not achieve clinical remission by week 24, and 28 had a relapse after achieving clinical remission. Cumulative rates of the events of poor treatment outcomes in patients with and without LVLs were 47.5% and 17.7%, respectively. A multivariable model showed the presence of LVLs at baseline was significantly associated with poor treatment outcomes (adjusted hazard ratio [HR] 3.54, 95% CI 1.52–8.24, *p* = 0.003). Cranial lesions and PMR did not increase the risk of poor treatment outcomes.

**Conclusion:**

The initial treatment intensity in the treatment algorithm of GCA could be determined based upon the presence or absence of LVLs detected by imaging at baseline.

## Background

Giant cell arteritis (GCA) is a vasculitis syndrome that usually develops in patients aged 50 years and over. GCA is characterized by cranial symptoms due to lesions in the superficial temporal artery, the maxillary artery, and the ophthalmic artery, and large vessel lesions (LVLs) in the aorta or its branches. An epidemiological study from a Japanese nationwide survey in 1998 showed a lower prevalence of GCA in Japan than in Western countries and almost the same clinical features of GCA [1].

The subclavian artery is a key location for LVLs in GCA, and Brack et al. proposed the definition of large vessel GCA (LV-GCA) as subclavian artery vasculitis in aged populations [2]. Aortic aneurysm is also an important clinical feature of GCA that is related to mortality [3, 4]. Aortic aneurysm is more complicated in GCA patients than in the general population [5]. Modified American College of Rheumatology (ACR) GCA classification criteria proposed by the Vasculitis Clinical Research Consortium [6] included LVLs as typical clinical findings of GCA [7], and more recently, in line with the evolution of imaging diagnosis of LVLs [8, 9], wall thickening by enhanced CT or MRI or ^18^fluorodeoxyglucose (FDG) uptake in the LVLs by FDG positron emission tomography (PET) is recognized to be common clinical findings of GCA [8, 10].

High dose of glucocorticoids (GCs) monotherapy is a standard induction regimen of GCA, with the rapid resolution of signs and symptoms of temporal arteritis and polymyalgia rheumatica (PMR) [11], but previous observational studies reported that the disease worsened in 30–50% of the patients during tapering of GCs [6, 12, 13], and long-term GC therapy is required to prevent relapses of the disease. Increasing cumulative GC dose is associated with drug-related adverse events (AEs) [14], and treatment strategy that is able to reduce the cumulative dose of GCs is valuable [11, 15]. Recent studies tried to resolve the unmet medical needs of the treatment of GCA [16–18], and the efficacy of tocilizumab is clearly demonstrated in terms of achievement of sustained clinical remission without GC. In the new era of the treatment, identification of associated factors for treatment response to conventional immunosuppressive therapy without biologics may help to guide the precision medicine of GCA [11].

Thus, we assessed treatment outcomes of conventional immunosuppressive therapy without biologics, based on the pre-defined criteria of clinical remission and relapse. This is the first study in Japan by using data of the multi-center retrospective cohort of GCA including patients diagnosed with imaging findings of LVLs, which identified clinical findings at baseline associated with poor treatment outcomes during 52 weeks.

## Methods

### Database

Twenty-three university hospitals and referring hospitals with sufficient experience treating vasculitides participated in this retrospective multi-center study. All investigators were members of the Japan Research Committee of the Ministry of Health, Labour, and Welfare for Intractable Vasculitis (JPVAS). All incident cases of clinically diagnosed GCA from 2007 to 2014 who had started GC therapy were enrolled in the JPVAS large vessel vasculitis (LVV) cohort. The patients were diagnosed in each facility based on temporal arterial biopsy (TAB), 1990 ACR GCA classification criteria, and imaging findings of LVL based on the modified ACR GCA classification criteria [6]. The definition of clinical remission and relapse was determined by the study group before the start of the data collection in 2014. Investigators at each participating facility examined the medical records retrospectively and assessed clinical remission and relapse based on the pre-defined criteria. Data for 2 years after starting GCs were collected, using a pre-defined case report form at 4, 8, 24, 52, 76, and 104 weeks after the start of treatment. One-year outcomes were examined in the present study. Investigators at each participating facility reported the date of achievement of clinical remission, disease status at these time points, date of relapse, and clinical manifestations at the relapse including imaging findings and treatment intensification. The definition of clinical remission and relapse is described below.

### Outcomes

The primary endpoint was an event of poor treatment outcomes (non-achievement of clinical remission by week 24 or relapse after achieving clinical remission during 52 weeks). Clinical remission was defined as the absence of active disease. The active disease at baseline was defined as having clinical signs and symptoms in the cranial and large vessel areas (Additional file: Table S1) or PMR, and/or elevation of C-reactive protein (CRP) levels. We discriminated persisted ischemic signs and symptoms (i.e., visual manifestations or claudication) from the damage of GCA at week 24 after the start of treatment as follows: if the signs and symptoms of active disease at baseline persisted without worsening for 6 months or longer and CRP level was normalized, these were not considered as clinical findings of active disease at week 24. A patient was determined to have a relapse if he/she had either one of the signs and symptoms that newly appeared or worsened after the achievement of clinical remission, or deterioration of imaging findings of LVL. Re-elevation of CRP level above the laboratory normal limit (CRP < 0.3 mg/dl) without clinical manifestations was considered as a relapse only if other causes such as infection were excluded and the treatment was intensified.

### Confirmation of relapse by the study group

Three investigators (HAU, HY, YN) independently reviewed the clinical findings at the relapse, treatment intensification, and clinical course after the relapse in a manner to blind imaging data at baseline about LVL, and confirmed whether the data met the definition of relapse in the present study, especially, in the relapse cases who did not have clinical signs and symptoms of active disease and showed an elevated CRP level. Finally, one patient was judged as infection but not relapse, because the patient was diagnosed with miliary tuberculosis after the intensification of the treatment.

### Safety

Safety was assessed by collecting information for 0–52 weeks after the start of treatment. This information included the development of serious infections, fractures, cardiovascular lesions requiring hospitalization or prolonged hospitalization, cerebrovascular lesions requiring hospitalization or prolonged hospitalization, gastrointestinal bleeding or perforation requiring hospitalization or prolonged hospitalization, diabetes mellitus requiring drug therapy, glaucoma or a cataract requiring drug therapy or eye surgery, psychiatric symptoms requiring drug therapy, and death.

### Collection of imaging data of LVL

The site investigators reported wall thickening, wall edema, stenosis, aneurysm, or dissection in anatomical sites of lesions of the aortic branches (carotid, vertebral, brachiocephalic, subclavian, axillary, pulmonary, renal, and iliac/femoral arteries) and aortic lesions (ascending aorta, aortic arch, thoracic descending aorta, and abdominal aorta) based on the results of at least one of computed tomography (CT), CT angiography (CTA), magnetic resonance imaging (MRI), or magnetic resonance angiography (MRA). Data were also collected on FDG uptake in the arterial wall using FDG-PET. Information about lesions of cerebral, basilar, coronary, and mesenteric arteries was also collected.

### Statistics

Student’s *t* test and the Mann-Whitney test were used to compare continuous variables depending on their distribution, and the chi-squared test and Fisher’s exact test were used for categorical variables. The patients who dropped out within 24 weeks after treatment were excluded from the analysis of clinical remission and relapse. In the analysis of poor treatment outcomes, patients who did not achieve clinical remission by week 24 were deemed to have had an event of poor treatment outcome at week 0 as described in the previous study [18]. Patients with relapse after the achievement of clinical remission were considered to have had an event at the date of relapse. Univariable and multivariable analyses for associated factors with the event of poor treatment outcomes during 52 weeks were conducted by using the Cox proportional hazard model. The Omnibus test was used as the goodness-of-fit test. Cumulative rates and median time of the first event of poor treatment outcomes during 52 weeks were analyzed using the Kaplan-Meier method and the log-rank test. A sensitivity analysis was implemented by using relapse after the achievement of clinical remission as an event. All analyses were performed using SPSS version 21 (IBM, Armonk, NY, USA). All reported *p* values are two-tailed, and the level of significance is *p* < 0.05.

## Results

### Patient characteristics

Case report forms of 139 newly diagnosed patients were assessed (Table 1). All patients (*n* = 139) were Asian. The mean (SD) age was 73.8 (7.7) years, mean weight (SD) was 50.9 (10.4) kg, and 66.9% were women. The age of onset was 70 and older in 98 patients, 60–69 in 38, and 50–59 in 3. The median disease duration was 3.1 months (interquartile range [IQR] 1.6–6.3). Overall, 108 (77.7%) of the 139 had clinical signs and symptoms of cranial lesions, 36 (25.9%) clinical signs and symptoms of LVLs, and 58 (41.7%) PMR. TAB was conducted in 86 patients, and 70 (50.4%) of the 139 patients were biopsy-proven GCA. Imaging examinations of LVLs were performed in 135 (97.1%) patients, and 97 received enhanced CT or CT angiography, 50 enhanced MRI or MR angiography, 31 carotid ultrasonography, 61 cardiac sonography, 14 non-enhanced CT, 17 non-enhanced MRI, and 38 PET-CT. LVLs were detected in 73 (52.5%) of the 139 patients. Thirty-five of the 73 patients with LVLs were also confirmed with data on PET-CT. Imaging data were summarized in Table 2. Lesions of the subclavian artery, any aortic branches, or aorta were found in 42 (57.5%), 54 (74.0%), or 50 (68.5%) of the 73 patients, respectively, and stenosis of the subclavian artery or aortic aneurysm was observed in 14 (19.2%) or 7 (9.6%), respectively. Any vascular damage of the large vessels by imaging data was observed in 31 (42.5%) of the 73 patients. Sixteen (38.1%) of the 42 patients with lesions of the subclavian arteries detected by imaging data had clinical signs and symptoms of the upper limbs (claudication, decreased or absent radial pulse, or blood pressure asymmetry > 10 mmHg). One had pulmonary hypertension due to stenosis of the pulmonary artery. Annuloaortic ectasia and aortic insufficiency caused by vasculitis were not reported.

**Table 1:** Demographic and clinical features of the giant cell arteritis cohort at baseline

Characteristics	Value
Age, years, mean ± SD (*n* = 139)	73.8 ± 7.7
Female patients, % (*n* = 139)	66.9
Weight, kg, mean ± SD (*n* = 139)	50.9 ± 10.4
GCA ACR classification criteria, % (*n* = 139)	78.4
Modified GCA classification criteria, % (*n* = 139)	99.3
TAB performed, % (*n* = 139)	61.9
TAB positive, % (*n* = 139)	50.4
Imaging performed, % (*n* = 139)	96.6
Imaging positive, % (*n* = 139)	52.5
Signs and symptoms of cranial lesions, % (*n* = 139)	77.7
Headache, % (*n* = 139)	61.2
Abnormal temporal artery, % (*n* = 139)	59.0
Jaw claudication, % (*n* = 139)	36.0
Visual disturbance, % (*n* = 139)	23.7
Visual loss, % (*n* = 139)	4.3
Signs and symptoms^a^ of LVL, % (*n* = 139)	25.9
Neck, % (*n* = 136)	10.3
Upper limbs, % (*n* = 135)	11.8
Lower limbs, % (*n* = 133)	3.0
Chest or abdominal bruit, % (*n* = 131)	9.2
Fever, % (*n* = 137)	32.1
Constitutional symptoms, % (*n* = 132)	75.8
Polymyalgia rheumatica, % (*n* = 139)	41.7
CRP, mg/dl, median (interquartile range) (*n* = 139)	7.2 (3.3–11.2)
Albumin, mg/dl, mean ± SD (*n* = 124)	3.1 ± 0.6
Ischemic heart disease, % (*n* = 138)	7.2
Cerebrovascular disease, % (*n* = 135)	14.1
Chronic lung disease, % (*n* = 137)	8.0
Hypertension, % (*n* = 138)	44.9
Diabetes mellitus, % (*n* = 138)	21.0
Hyperlipidemia, % (*n* = 138)	25.3
Osteoporosis, % (*n* = 121)	21.5
Dementia, % (*n* = 137)	1.5

*LVL* large vessel lesions, *LV* large vessel, *ACR* American College of Rheumatology, *CRP* C-reactive protein

^a^Information about any signs and symptoms of LVL was reported in all enrolled patients. Signs and symptoms of the neck included tenderness of the carotid arteries, carotid bruit, or neck claudication. Signs and symptoms of the upper limb included arm claudication, decreased or absent radial pulse, or blood pressure asymmetry > 10 mmHg. Lower limb included leg claudication or decreased or absent pulse of lower limb

**Table 2:** Imaging findings in the patients with LVL (*n* = 73)

	Any imaging findings^a^	Wall thickening, wall edema, or FDG uptake	Stenosis	Aneurysm
Left carotid, %	41.1	37.0	8.2	0
Right carotid, %	32.9	31.5	2.7	0
Vertebral, %	8.2	5.5	6.8	0
Brachiocephalic, %	31.5	30.1	2.7	0
Left subclavian, %	53.4	46.6	11.0	1.4
Right subclavian, %	43.8	39.7	8.2	0
Left axillary, %	20.5	16.4	5.5	0
Right axillary, %	16.4	15.1	0	1.4
Pulmonary, %	1.4	0	1.4	0
Coronary, %	2.6	0	2.6	0
Ascending aorta, %	31.5	28.8	0	4.1
Aortic arch, %	47.9	47.9	0	2.7
Descending thoracic aorta, %	49.3	47.9	0	0
Abdominal aorta, %	53.4	53.4	0	2.7
Renal, %	6.8	2.7	2.7	1.4
Hepatic, %	1.4	0	0	1.4
Mesenteric, %	1.4	0	1.4	0
Iliac or femoral artery, %	19.2	16.4	6.8	1.4

Imaging findings were collected from 139 enrolled patients

*LVL* large vessel lesions, *FDG*^18^fluorodeoxyglucose

^a^Any imaging findings include wall thickening, wall edema, arterial FDG uptake, stenosis, or aneurysms

### Diagnosis of GCA

Overall, 30 (21.6%) of the 139 patients were diagnosed by both positive TAB and positive imaging, 40 (28.8%) by positive TAB, and 43 (30.9%) by positive imaging based on the modified ACR GCA classification criteria [6]. Twenty-six (18.7%) did not have positive TAB or positive imaging and were diagnosed based on the 1990 ACR GCA classification criteria.

### Clinical remission by week 24

**Fig. 1:**
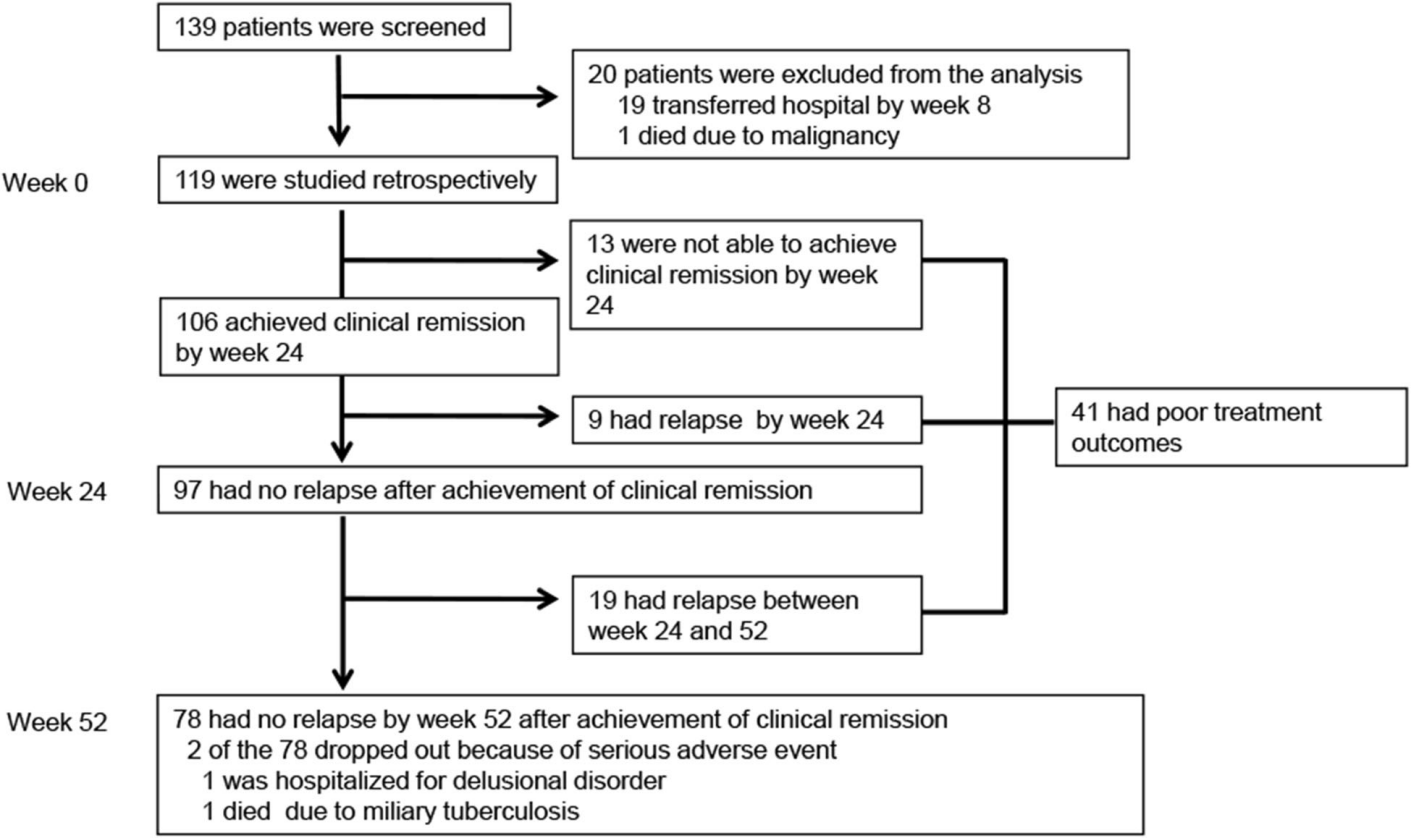
Screening, follow-up of the patients, and treatment outcomes. Clinical remission and relapse were evaluated in 119 newly diagnosed patients who were observed for more than 24 weeks. Thirteen had worsening of clinical signs and symptoms or persisted elevation of CRP and were not able to achieve clinical remission. Nine had a relapse after the achievement of clinical remission between weeks 0 and 24, and 97 had no relapse at week 24 after the achievement of clinical remission. Nineteen of the 97 had relapse between weeks 24 and 52, and 78 had no relapse at week 52 after the achievement of clinical remission. Overall, 41 had poor treatment outcomes

Of the 139 newly diagnosed patients with GCA, clinical remission was evaluated in 119 (85.6%) patients who were observed for 24 weeks or longer, and 20 patients were excluded from the analysis (Fig. 1). Patient characteristics of the 119 patients were the same as those of the 139 patients of the entire cohort (Additional file: Table S2).

The initial mean dose (SD) of prednisolone (PSL) was 0.76 (0.23) mg/kg/day, and 35 (29.4%) of the 119 patients received any concomitant immunosuppressive drugs for induction therapy. Methotrexate (MTX) was administered for the initial treatment in 20 patients, azathioprine (AZA) in 11, and cyclophosphamide (CY) in 4. No patients received biologic agents. Headache and/or abnormal temporal artery was observed in 87 patients at baseline and improved at week 4 in 84 (96.6%) and disappeared in all patients after week 8. Jaw claudication was found in 45 patients at baseline and disappeared in 44 (97.8%) by week 24. Twenty patients had persisted signs and symptoms without worsening at week 24 (visual manifestations 12, neck 1, and upper limb 7), and CRP levels were normalized in all 20 patients. Thus, the 20 patients were deemed not to have active disease at week 24. A total of 106 patients achieved clinical remission by week 24 (Fig. 1).

Clinical courses of the thirteen patients without clinical remission (i.e., worsening of clinical signs and symptoms or persisted elevation of CRP throughout 24 weeks) were as follows: one dissection and rupture of ascending aorta and post-cardiac arrest encephalopathy 11 days after treatment, one worsening of claudication of the lower limbs at week 24, one progression of stenosis of the vertebral artery at week 24, one acute heart failure and post-cardiac arrest encephalopathy at week 52, one deterioration of claudication of lower legs at week 24, one deterioration of jaw claudication at week 24, six subclinical arteritis of LVLs with persisted elevation of CRP, and one subclinical arteritis of cranial lesions with persisted elevation of CRP.

### Relapse after achievement of clinical remission

Relapse after the achievement of clinical remission was reported in a total of 28 patients: 9 between weeks 0 and 24 and 19 between weeks 24 and 52 (Fig. 1). The mean time to relapse of patients was 307 days (95% CI 283–332). The median dose of PSL at the time of relapse was 8.0 mg/day (IQR 5–10). Cranial signs and symptoms were reported in 5 (17.9%) of the 28 relapse patients, headache in four, jaw claudication in one, and visual disturbance in zero. PMR was reported in five (17.9%) and constitutional symptoms in four (14.3%). Clinical signs and symptoms of LVLs were not observed at the relapse. The elevation of CRP without clinical signs and symptoms followed by treatment intensification was reported in 14 (50.0%) of the 28 relapse patients. Four of the 14 patients showed deterioration of imaging findings of LVLs with the following clinical course: one progression of stenosis of the left subclavian and ascending aorta, one dissection of descending thoracic aorta, one expansion of the abdominal aorta, and one expansion of the right subclavian artery. Eight of the 14 patients had a relapse of subclinical LVLs with re-elevation of CRP, and two had subclinical cranial lesions with re-elevation of CRP. Overall, PSL dose was increased in 11 patients, and immunosuppressants were added in 18 patients of the 28 patients with relapse. MTX was started in 12 patients, AZA in 2, tacrolimus in 3, and CY in 1. Two patients with clinical signs and symptoms of active disease at the relapse did not have information about treatment intensification.

### Clinical characteristics of patients with the event of poor treatment outcomes

Overall, 41(34.5%) of the 119 patients had the event of poor treatment outcomes (i.e., non-achievement of clinical remission by week 24 or relapse after achieving remission during 52 weeks) (Fig. 1). Two of the 78 patients without the event of poor treatment outcomes dropped out because of serious adverse events (AEs). Patients with the event of poor treatment outcomes had less cranial signs and symptoms and more LVLs, especially aortic lesions, at baseline detected by imaging examinations (Table 3). Frequency of PMR and comorbidities, median CRP at baseline, mean weight, and mean PSL doses at weeks 0, 4, 8, and 12 were similar between the two groups.

**Table 3:** Clinical characteristics of patients with and without the event of poor treatment outcomes

	With the event^a^ (*n* = 41)	Without the event^b^ (*n* = 78)	*p*
Age, years, mean ± SD	72.6 ± 8.3	73.3 ± 7.3	0.622
Female patients, %	73.2	64.1	0.317
Weight, kg, mean ± SD	49.4 ± 9.2	52.5 ± 11.1	0.128
Signs and symptoms of cranial lesions at baseline, %	65.9	83.3	0.03
Headache, %	51.2	64.1	0.173
Abnormal temporal artery, %	51.2	64.1	0.173
Jaw claudication, %	36.6	32.1	0.619
Visual disturbance, %	19.5	25.6	0.454
LVLs detected by imaging at baseline, %	78.0	46.2	0.001
Any lesions of the aortic branches^c^, %	48.8	37.2	0.222
Any lesions of the aorta^d^, %	53.7	30.8	0.015
Any structural vascular damage^e^, %	31.7	19.2	0.127
Stenosis of the aortic branches^c^, %	12.2	15.4	0.637
Aneurysm of the aorta, %	9.8	2.6	0.106
Fever at baseline, %	24.4	36.8	0.170
Polymyalgia rheumatica at baseline, %	43.9	41.0	0.763
CRP at baseline, mg/dl, median (interquartile range)	7.15 (3.75–11.1)	6.82 (2.99–11.2)	0.810
Ischemic heart disease at baseline, %	2.4	7.7	0.235
Cerebrovascular disease at baseline, %	17.1	11.5	0.400
Hypertension at baseline, %	39.0	44.9	0.540
Diabetes mellitus at baseline, %	19.5	17.9	0.835
Hyperlipidemia at baseline, %	22.0	29.5	0.378
Initial dose of PSL, mg/kg/day, mean ± SD	0.76 ± 0.26	0.75 ± 0.22	0.799
PSL dose at week 4, mg/kg/day, mean ± SD	0.57 ± 0.20	0.56 ± 0.16	0.652
PSL dose at week 8, mg/kg/day, mean ± SD	0.42 ± 0.16	0.41 ± 0.14	0.774
PSL dose at week 12, mg/kg/day, mean ± SD	0.36 ± 0.15	0.34 ± 0.11	0.364
Immunosuppressive drug use at baseline, %	15.0	9.0	0.245
Immunosuppressive drug use during observational period, %	75.6	28.2	< 0.001
MTX for induction therapy, *n* (%)	11 (26.8)	9 (11.5)	–
MTX for flare, %	12 (29.3)	–	–
CY for induction therapy, %	2 (4.9)	2 (2.6)	–
CY for flare, %	3 (7.3)	–	–
AZA for induction therapy, %	7 (17.1)	4 (5.1)	–
AZA for flare, %	2 (4.9)	–	–

*LVLs* large vessel lesions, *LV* large vessel, *ACR* American College of Rheumatology, *CRP* C-reactive protein, *PSL* prednisolone, *GCs* glucocorticoids, *MTX* methotrexate, *CY* cyclophosphamide, *AZA* azathioprine

^a^Poor treatment outcomes were defined as non-achievement of clinical remission by week 24 or relapse after the achievement of clinical remission during 52 weeks

^b^Patients without the event of poor treatment outcomes achieved clinical remission by week 24 and no relapse during 52 weeks.

^c^Any lesions of aortic branches by imaging included lesions in the carotid, vertebral, brachiocephalic, subclavian, axillary artery, pulmonary, renal, or iliac arteries

^d^Any lesions of the aorta by imaging included lesions in the ascending aorta, aorta arch, descending thoracic aorta, or abdominal aorta

^e^Any structural vascular damage included stenosis, dilatation, or aneurysm in lesions of the aortic branches and aorta

### Associated factors of the event of poor treatment outcomes in patients with GCA

**Fig. 2:**
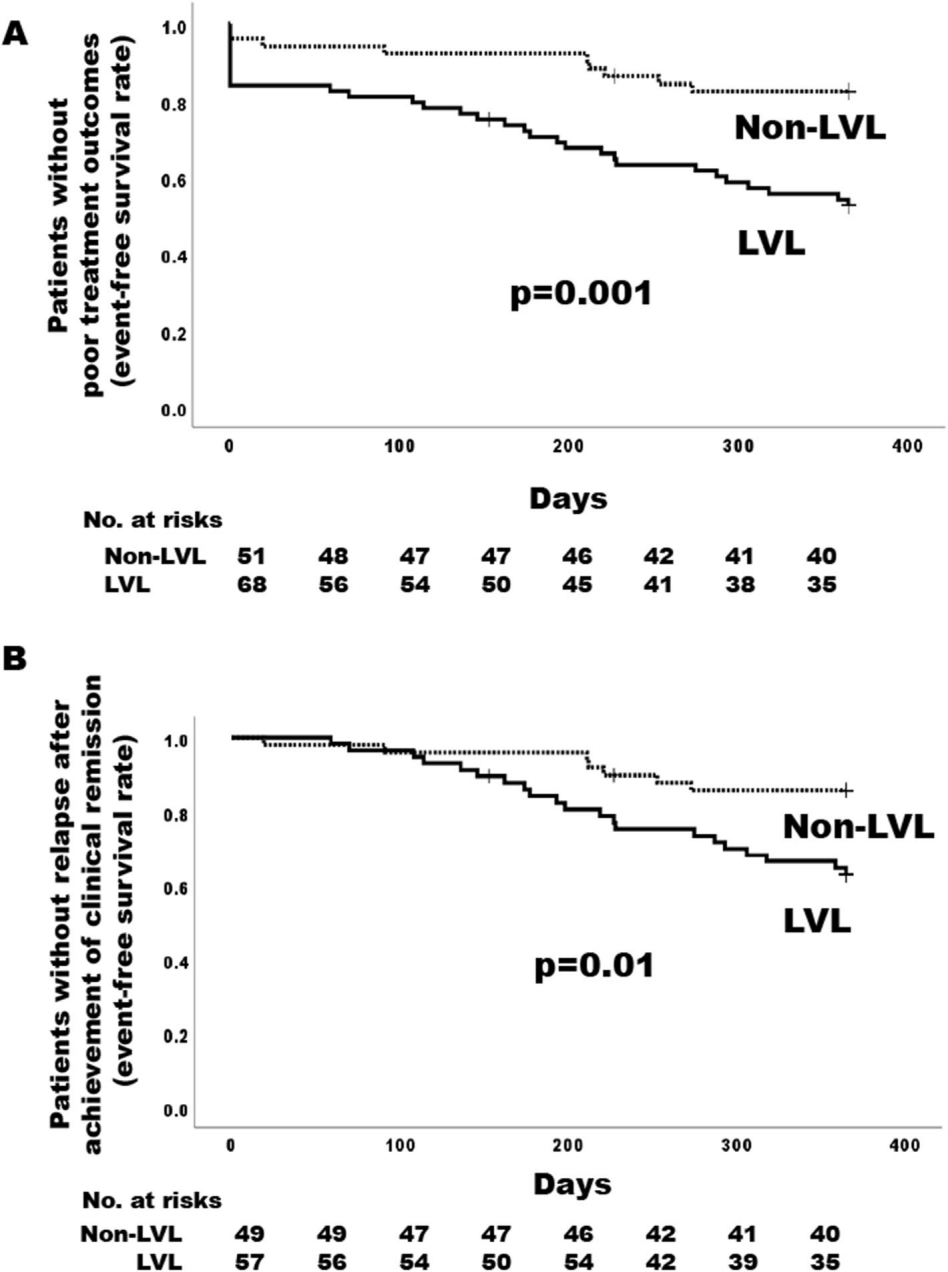
Event-free curve in patients who were observed for 24 weeks or longer. **a** Time to poor treatment outcomes analyzed in all 119 patients who were observed for 24 weeks or longer. Patients who did not achieve clinical remission by week 24 were considered to have had an event at week 0. **b** Time to the first relapse in 106 patients who achieved clinical remission by week 24

Associated factors of the event of poor treatment outcomes were analyzed for the 119 patients by using the Cox proportional hazard model as described in the “Methods” section. Univariable Cox proportional hazard model showed the LVL was significantly associated with the event of poor treatment outcomes (hazard ratio [HR] 3.20, 95% CI 1.53–6.72, *p* = 0.002). Interestingly, among LVLs, the presence of any lesions of the aorta (HR 2.07, 95% CI 1.12–3.82, *p* = 0.02) was a significant factor, while the presence of any lesions of the aortic branches (HR 1.44, 95% CI 0.78–2.66, *p* = 0.240) was not (Table 4). Any stenosis of the aortic branches was not associated with the event of poor treatment outcomes, but any aortic aneurysm increased the risk of the event numerically. Age; sex; presence of PMR; presence of any comorbidities; CRP levels at baseline; PSL doses at weeks 0, 4, 8, and 12; and immunosuppressive drug use at baseline were not significantly associated factors for the event of poor treatment outcomes. The presence of any cranial lesions significantly reduced the risk of the event. The mean time to the event of patients with LVLs (258 days, 95% CI 224–292) was significantly shorter than that of patients without LVLs (326 days, 95% CI 299–352) by log-rank test (*p* = 0.001) (Fig. 2a). The cumulative rate of the event of poor treatment outcomes in patients with and without LVLs was 47.5% and 17.7%, respectively.

**Table 4:** Associated factors with poor treatment outcomes during 52 weeks

	Univariable analysis		Multivariable analysis^a^	
	HR (95% CI)	*p*	HR (95% CI)	*p*
Age, per 1 year increment	0.99 (0.95–1.03)	0.836	1.02 (0.97–1.08)	0.388
Female	1.44 (0.72–2.88)	0.396	1.28 (0.63–2.62)	0.492
Any cranial symptoms at baseline	0.50 (0.26–0.95)	0.034	0.83 (0.40–1.72)	0.622
Polymyalgia rheumatica at baseline	1.13 (0.61–2.09)	0.699	1.30 (0.63–2.62)	0.492
LVLs at baseline	3.20 (1.53–6.72)	0.002	3.54 (1.52–8.24)	0.003
Any lesions of the aortic branches^b^	1.44 (0.78–2.66)	0.240		
Any lesions of the aorta^c^	2.07 (1.12–3.82)	0.02		
Any structural vascular damage^d^	1.73 (0.90–3.35)	0.102		
Aneurysm of the aorta	2.76 (0.98–7.78)	0.054		
CRP at baseline per 1 mg/dl increment	1.00 (0.95–1.05)	0.930		
Initial dose of PSL per 0.1 mg/kg/day increment	1.01 (0.88–1.17)	0.874		
Dose of PSL at week 4 per 0.1 mg/kg/day increment	1.04 (0.86–1.25)	0.685		
Dose of PSL at week 8 per 0.1 mg/kg/day increment	1.03 (0.83–1.29)	0.785		
Dose of PSL at week 12 per 0.1 mg/kg/day increment	1.13 (0.87–1.46)	0.356		
Immunosuppressive drug use at baseline	1.54 (0.65–3.67)	0.330		

Poor treatment outcomes were defined as non-achievement of clinical remission by week 24 or relapse after the achievement of clinical remission during 52 weeks

*LVLs* large vessel lesions, *CRP* C-reactive protein, *PSL* prednisolone

^a^Age, sex, any cranial symptoms, and polymyalgia rheumatica were selected as covariates of interest

^b^Any lesions of the aortic branches by imaging included lesions in the carotid, vertebral, brachiocephalic, subclavian, axillary artery, pulmonary renal, or iliac arteries

^c^Any lesions of the aorta by imaging included lesions in the ascending aorta, aorta arch, descending thoracic aorta, or abdominal aorta

^d^Any structural vascular damage included stenosis, dilatation, or aneurysm in lesions of the aortic branches and aorta

In a multivariable analysis, age, sex, any cranial lesions, and PMR were selected as covariates of interest, and the presence of the LVL was only the significantly associated factor of the event of poor treatment outcomes during 52 weeks (adjusted HR 3.53, 95% CI 1.52–8.24, *p* = 0.003) (Table 4).

### Associated factors of relapse in patients who achieved clinical remission

Relapse was evaluated in 106 patients who achieved clinical remission by week 24. In univariable analysis, associated factors with relapse were similar to those with the event of poor treatment outcomes (Additional file: Table S3). The presence of any aortic lesions, but not any lesions of the aortic branches, was a significant factor of relapse. The mean time to relapse of patients with LVLs was significantly shorter than that of patients without LVLs (*p* = 0.01) (Fig. 2b). The cumulative rate of relapse after the achievement of clinical remission in patients with and without LVLs was 37.4% and 14.4%, respectively. Multivariable analysis showed that the presence of the LVL was only the significantly associated factor of relapse after the achievement of clinical remission during 52 weeks (adjusted HR 4.41, 95% CI 1.62–12.0, *p* = 0.004) (Additional file: Table S3).

### Safety

AEs during 52 weeks in the 119 patients are shown in Table S4 of additional file. Serious infections occurred in 9 (13.2%) of 68 LVL patients and in 10 (19.6%) of 51 non-LVL patients. Five patients died during the observational period. AE leading to death in the patients with LVLs were *Pneumocystis jirovecii* pneumonia and cerebral infarction, ischemic heart disease after dissection of the descending thoracic aorta, and sudden death of unknown origin after the expansion of abdominal aorta in one patient for each, and those in the patients without LVLs were miliary tuberculosis and sudden death in one patient for each. Cerebrovascular event was reported (*n* = 1) but was unclear due to vasculitis.

## Discussions

Almost 35% of the patients with GCA had the event of poor treatment outcomes during 52 weeks in the multi-centric retrospective cohort study in Japan; 11% was not able to achieve clinical remission by week 24, and 24% had a relapse after the achievement of clinical remission. We have demonstrated that the presence of LVLs detected by imaging studies at baseline was significantly associated with the event of poor treatment outcomes. A sensitivity analysis using relapse alone as an event also showed association with LVLs. Interestingly, among LVLs, any aortic lesions are more likely to be associated with the event of poor treatment outcomes. These data suggest that GCA patients with LVLs, especially aortic lesions, may be resistant to initial conventional immunosuppressive therapy without biologics.

The present study showed that the mean age at diagnosis, male to female ratio, frequency of cranial signs and symptoms, and PMR were almost the same as epidemiological findings in Western countries [7, 13, 19–21]. Prevalence and distribution of LVLs were various among previous cohorts [4, 19, 20, 22–25] by different inclusion criteria and imaging modalities used for the diagnosis. In our cohort, LVLs were detected in about half of GCA patients, and one fifth of the patients were diagnosed as having GCA using LV imaging without satisfying the 1990 GCA ACR classification criteria. These are almost the same as a recent RCT, which adopted the eligibility criteria including imaging for recruitment of active patients [18, 19, 26]. The distribution of LVLs in our study was almost similar to recent studies of GCA with LVLs [23, 27].

Serious outcomes, such as dissection and rupture of aneurysms, were reported in previous studies [28–30]. Progressive vascular damage of LVLs during 52 weeks was reported in six (8.8%) of the 68 patients with LVLs in the present study, and these values were almost the same as previous reports [20, 22].

The evaluation of disease activity and vascular damage of LVV is challenging [31–33]. Ocular manifestations and large artery complications were common damages in patients with GCA [34]. In our study, upper limb claudication and visual disturbance that persisted without worsening for 6 months or longer were deemed as an inactive disease at week 24. However, the imaging progression of LVLs might be underestimated in the present study, because follow-up imaging studies were not conducted in all of the patients.

Treatment responses of GCA with LVLs are controversial among observational studies [20, 21, 35–38]. The present study showed only the presence of LVLs at baseline was an associated factor of non-achievement of clinical remission or relapse, while initial PSL dose, PSL tapering speed during the initial 12 weeks, and immunosuppressive drug use at baseline were not. However, associated factors of relapse or severe complications were various (i.e., age, hypertension, diabetes at GCA diagnosis, ischemic heart disease, cranial signs and symptoms, CRP at baseline, and dose of PSL at baseline) [13, 30, 38–41]. Differential treatment response and discrepancy of the results in each cohort may be influenced by the definition of clinical remission and relapse, prevalence of LVLs, and severity of LVLs at baseline.

CRP elevation without clinical signs and symptoms was not a reliable indicator of relapse of cranial lesions and PMR [42], while clinical signs and symptoms of active GCA were not necessarily observed in patients with progression of LVLs [22, 36, 43–46]. Active LVLs of GCA without clinical signs and symptoms were demonstrated by PET-CT [47, 48]. Previous studies also showed CRP elevation without clinical signs and symptoms was a common finding during tapering of PSL dose [42] or at relapse of the disease in GCA cohorts [13, 17]. The present study also showed that elevation or re-elevation of CRP without clinical signs and symptoms was a common finding in the active GCA with LVLs at baseline or relapse. The reason for treatment intensification reported in 43% of the relapsed cases was the elevated CRP and/or vascular structural progression, which were considered as the residual vascular inflammation of LVLs without clinical signs and symptoms.

There are some limitations associated with the retrospective design of this study. First, selection bias due to retrospective design should be considered, but the risk is small because suspected patients with GCA who were referred to the participating hospitals of this study were identified using data from electronic medical records, and the investigators enrolled all cases. Second, 20 patients were excluded from the outcome analysis due to the short follow-up period. But these patients had no relapse and did not impact the interpretation of the data. Third, a uniform assessment of LVL at baseline and relapse, especially PET-CT, which was not covered by health insurance before 2018 in Japan, was not implemented for all the patients. However, imaging might be rather more frequently examined for diagnosis of GCA in Japan than in other countries, and the frequency of LVL at baseline was as expected based on previous reports [19, 20]. Fourth, the drug therapy was selected at the discretion of an attending physician, and the present study was not able to interpret the optimal protocol of glucocorticoid therapy and the benefit of immunosuppressive drugs for GCA. Fifth, 139 newly diagnosed patients were enrolled from 23 facilities, and the number of patients per facility may be fewer than previous studies in Western countries. However, the number of enrolled patients was higher than expected, since the prevalence rate of Japanese GCA patients was much lower than in Western countries [1].

## Conclusions

LVLs are detected by imaging in about half of the Japanese patients at baseline in the present study. LVLs, especially any aortic lesions, were an associated factor for poor treatment outcomes in the conventional immunosuppressive therapy without biologics. Initial treatment intensity could be determined based upon the presence or absence of LVLs. Our research results will be important for establishing a future treatment strategy for GCA.

## Supplementary information

**Additional file 1.** Supplementary tables.

## Data Availability

All of the data supporting the conclusions of this article are included within the article.

## Abbreviations

ACR: American College of Rheumatology
AEs: Adverse events
AZA: Azathioprine
CRP: C-reactive protein
CY: Cyclophosphamide
FDG: ^18^Fluorodeoxyglucose
GCA: Giant cell arteritis
GCs: Glucocorticoids
JPVAS: Japan Research Committee of the Ministry of Health, Labour, and Welfare for Intractable Vasculitis
LVLs: Large vessel lesions
LV: Large vessel
MTX: Methotrexate
PET: Positron emission tomography
PMR: Polymyalgia rheumatica
PSL: Prednisolone
TAB: Temporal arterial biopsy

## References

[1] Kobayashi S, Yano T, Matsumoto Y, Numano F, Nakajima N, Yasuda K (2003). Clinical and epidemiologic analysis of giant cell (temporal) arteritis from a nationwide survey in 1998 in Japan: the first government-supported nationwide survey. Arthritis Rheum.

[2] Brack A, Martinez-Taboada V, Stanson A, Goronzy JJ, Weyand CM (1999). Disease pattern in cranial and large-vessel giant cell arteritis. Arthritis Rheum.

[3] Nuenninghoff DM, Hunder GG, Christianson TJ, McClelland RL, Matteson EL (2003). Mortality of large-artery complication (aortic aneurysm, aortic dissection, and/or large-artery stenosis) in patients with giant cell arteritis: a population-based study over 50 years. Arthritis Rheum.

[4] Kermani TA, Warrington KJ, Crowson CS, Ytterberg SR, Hunder GG, Gabriel SE (2013). Large-vessel involvement in giant cell arteritis: a population-based cohort study of the incidence-trends and prognosis. Ann Rheum Dis.

[5] Robson JC, Kiran A, Maskell J, Hutchings A, Arden N, Dasgupta B (2015). The relative risk of aortic aneurysm in patients with giant cell arteritis compared with the general population of the UK. Ann Rheum Dis.

[6] Kermani TA, Warrington KJ, Cuthbertson D, Carette S, Hoffman GS, Khalidi NA (2015). Disease relapses among patients with giant cell arteritis: a prospective, longitudinal cohort study. J Rheumatol.

[7] Hunder GG, Bloch DA, Michel BA, Stevens MB, Arend WP, Calabrese LH (1990). The American College of Rheumatology 1990 criteria for the classification of giant cell arteritis. Arthritis Rheum.

[8] Dejaco C, Ramiro S, Duftner C, Besson FL, Bley TA, Blockmans D, et al. EULAR recommendations for the use of imaging in large vessel vasculitis in clinical practice. Ann Rheum Dis. 2018;77:636–43. 10.1136/annrheumdis-2017-212649 29358285

[9] Prieto-Gonzalez S, Depetris M, Garcia-Martinez A, Espigol-Frigole G, Tavera-Bahillo I, Corbera-Bellata M (2014). Positron emission tomography assessment of large vessel inflammation in patients with newly diagnosed, biopsy-proven giant cell arteritis: a prospective, case-control study. Ann Rheum Dis.

[10] Mukhtyar C, Guillevin L, Cid MC, Dasgupta B, de Groot K, Gross W (2009). EULAR recommendations for the management of large vessel vasculitis. Ann Rheum Dis.

[11] Hellmich B, Agueda A, Monti S, Buttgereit F, de Boysson H, Brouwer E, et al. 2018 update of the EULAR recommendations for the management of large vessel vasculitis. Ann Rheum Dis. 2020;79:19–30. 10.1136/annrheumdis-2019-215672 31270110

[12] Proven A, Gabriel SE, Orces C, O’Fallon WM, Hunder GG (2003). Glucocorticoid therapy in giant cell arteritis: duration and adverse outcomes. Arthritis Rheum.

[13] Labarca C, Koster MJ, Crowson CS, Makol A, Ytterberg SR, Matteson EL (2016). Predictors of relapse and treatment outcomes in biopsy-proven giant cell arteritis: a retrospective cohort study. Rheumatology (Oxford).

[14] Wilson JC, Sarsour K, Collinson N, Tuckwell K, Musselman D, Klearman M (2017). Serious adverse effects associated with glucocorticoid therapy in patients with giant cell arteritis (GCA): a nested case-control analysis. Semin Arthritis Rheum.

[15] Stone JH (2018). Foreword: clinical challenges of diagnosing and managing giant cell arteritis. Rheumatology (Oxford).

[16] Hoffman GS, Cid MC, Rendt-Zagar KE, Merkel PA, Weyand CM, Stone JH (2007). Infliximab for maintenance of glucocorticosteroid-induced remission of giant cell arteritis: a randomized trial. Ann Intern Med.

[17] Seror R, Baron G, Hachulla E, Debandt M, Larroche C, Puechal X (2014). Adalimumab for steroid sparing in patients with giant-cell arteritis: results of a multicentre randomised controlled trial. Ann Rheum Dis.

[18] Stone JH, Tuckwell K, Dimonaco S, Klearman M, Aringer M, Blockmans D (2017). Trial of tocilizumab in giant-cell arteritis. N Engl J Med.

[19] Tuckwell K, Collinson N, Dimonaco S, Klearman M, Blockmans D, Brouwer E (2017). Newly diagnosed vs. relapsing giant cell arteritis: baseline data from the GiACTA trial. Semin Arthritis Rheum.

[20] Muratore F, Kermani TA, Crowson CS, Green AB, Salvarani C, Matteson EL (2015). Large-vessel giant cell arteritis: a cohort study. Rheumatology (Oxford).

[21] de Boysson H, Daumas A, Vautier M, Parienti JJ, Liozon E, Lambert M (2018). Large-vessel involvement and aortic dilation in giant-cell arteritis. A multicenter study of 549 patients. Autoimmun Rev.

[22] de Boysson H, Liozon E, Lambert M, Parienti JJ, Artigues N, Geffray L (2016). 18F-fluorodeoxyglucose positron emission tomography and the risk of subsequent aortic complications in giant-cell arteritis: a multicenter cohort of 130 patients. Medicine (Baltimore).

[23] Prieto-Gonzalez S, Arguis P, Garcia-Martinez A, Espigol-Frigole G, Tavera-Bahillo I, Butjosa M (2012). Large vessel involvement in biopsy-proven giant cell arteritis: prospective study in 40 newly diagnosed patients using CT angiography. Ann Rheum Dis.

[24] Gonzalez-Gay MA, Garcia-Porrua C, Pineiro A, Pego-Reigosa R, Llorca J, Hunder GG (2004). Aortic aneurysm and dissection in patients with biopsy-proven giant cell arteritis from northwestern Spain: a population-based study. Medicine (Baltimore).

[25] Garcia-Martinez A, Hernandez-Rodriguez J, Arguis P, Paredes P, Segarra M, Lozano E (2008). Development of aortic aneurysm/dilatation during the followup of patients with giant cell arteritis: a cross-sectional screening of fifty-four prospectively followed patients. Arthritis Rheum.

[26] Unizony SH, Dasgupta B, Fisheleva E, Rowell L, Schett G, Spiera R (2013). Design of the tocilizumab in giant cell arteritis trial. Int J Rheumatol.

[27] Grayson PC, Maksimowicz-McKinnon K, Clark TM, Tomasson G, Cuthbertson D, Carette S (2012). Distribution of arterial lesions in Takayasu’s arteritis and giant cell arteritis. Ann Rheum Dis.

[28] Evans JM, Bowles CA, Bjornsson J, Mullany CJ, Hunder GG (1994). Thoracic aortic aneurysm and rupture in giant cell arteritis. A descriptive study of 41 cases. Arthritis Rheum.

[29] Nuenninghoff DM, Hunder GG, Christianson TJ, McClelland RL, Matteson EL (2003). Incidence and predictors of large-artery complication (aortic aneurysm, aortic dissection, and/or large-artery stenosis) in patients with giant cell arteritis: a population-based study over 50 years. Arthritis Rheum.

[30] Kermani TA, Warrington KJ, Crowson CS, Hunder GG, Ytterberg SR, Gabriel SE (2016). Predictors of dissection in aortic aneurysms from giant cell arteritis. J Clin Rheumatol.

[31] Sreih AG, Alibaz-Oner F, Kermani TA, Aydin SZ, Cronholm PF, Davis T (2017). Development of a core set of outcome measures for large-vessel vasculitis: report from OMERACT 2016. J Rheumatol.

[32] Aydin SZ, Direskeneli H, Sreih A, Alibaz-Oner F, Gul A, Kamali S (2015). Update on outcome measure development for large vessel vasculitis: report from OMERACT 12. J Rheumatol.

[33] Kermani TA, Cuthbertson D, Carette S, Hoffman GS, Khalidi NA, Koening CL (2016). The Birmingham Vasculitis Activity Score as a measure of disease activity in patients with giant cell arteritis. J Rheumatol.

[34] Kermani TA, Sreih AG, Cuthbertson D, Carette S, Hoffman GS, Khalidi NA (2018). Evaluation of damage in giant cell arteritis. Rheumatology (Oxford).

[35] de Boysson H, Liozon E, Lambert M, Dumont A, Boutemy J, Maigne G (2017). Giant-cell arteritis: do we treat patients with large-vessel involvement differently?. Am J Med.

[36] Garcia-Martinez A, Arguis P, Prieto-Gonzalez S, Espigol-Frigole G, Alba MA, Butjosa M, et al. Prospective long term follow-up of a cohort of patients with giant cell arteritis screened for aortic structural damage (aneurysm or dilatation). Ann Rheum Dis. 2014;73:1826–32. 10.1136/annrheumdis-2013-203322 23873881

[37] Czihal M, Piller A, Schroettle A, Kuhlencordt P, Bernau C, Schulze-Koops H (2015). Impact of cranial and axillary/subclavian artery involvement by color duplex sonography on response to treatment in giant cell arteritis. J Vasc Surg.

[38] de Boysson H, Lambert M, Liozon E, Boutemy J, Maigne G, Ollivier Y (2016). Giant-cell arteritis without cranial manifestations: working diagnosis of a distinct disease pattern. Medicine (Baltimore).

[39] Hocevar A, Rotar Z, Jese R, Semrl SS, Pizem J, Hawlina M (2016). Do early diagnosis and glucocorticoid treatment decrease the risk of permanent visual loss and early relapses in giant cell arteritis: a prospective longitudinal study. Medicine (Baltimore).

[40] Salvarani C, Della Bella C, Cimino L, Macchioni P, Formisano D, Bajocchi G (2009). Risk factors for severe cranial ischaemic events in an Italian population-based cohort of patients with giant cell arteritis. Rheumatology (Oxford).

[41] Salvarani C, Cimino L, Macchioni P, Consonni D, Cantini F, Bajocchi G (2005). Risk factors for visual loss in an Italian population-based cohort of patients with giant cell arteritis. Arthritis Rheum.

[42] Stone JH, Tuckwell K, Dimonaco S, Klearman M, Aringer M, Blockmans D (2019). Glucocorticoid dosages and acute-phase reactant levels at giant cell arteritis flare in a randomized trial of tocilizumab. Arthritis Rheumatol.

[43] Prieto-Gonzalez S, Garcia-Martinez A, Tavera-Bahillo I, Hernandez-Rodriguez J, Gutierrez-Chacoff J, Alba MA (2015). Effect of glucocorticoid treatment on computed tomography angiography detected large-vessel inflammation in giant-cell arteritis. A prospective, longitudinal study. Medicine (Baltimore).

[44] Muratore F, Kermani TA, Crowson CS, Koster MJ, Matteson EL, Salvarani C, et al. Large vessel dilatation in giant cell arteritis: a different subset of disease? Arthritis Care Res (Hoboken). 2017;70:1406–11. 10.1002/acr.23498 29266882

[45] Kermani TA, Diab S, Sreih AG, Cuthbertson D, Borchin R, Carette S, et al. Arterial lesions in giant cell arteritis: a longitudinal study. Semin Arthritis Rheum. 2019;48:707–13. 10.1016/j.semarthrit.2018.05.002 PMC6226363 29880442

[46] Espitia O, Neel A, Leux C, Connault J, Espitia-Thibault A, Ponge T (2012). Giant cell arteritis with or without aortitis at diagnosis. A retrospective study of 22 patients with longterm followup. J Rheumatol.

[47] Grayson PC, Alehashemi S, Bagheri AA, Civelek AC, Cupps TR, Kaplan MJ (2018). (18) F-fluorodeoxyglucose-positron emission tomography as an imaging biomarker in a prospective, longitudinal cohort of patients with large vessel vasculitis. Arthritis Rheumatol..

[48] Quinn KA, Ahlman MA, Malayeri AA, Marko J, Civelek AC, Rosenblum JS (2018). Comparison of magnetic resonance angiography and (18)F-fluorodeoxyglucose positron emission tomography in large-vessel vasculitis. Ann Rheum Dis.

