# Supplementary material for: Associated factors of poor treatment outcomes in patients with giant cell arteritis: clinical implication of large vessel lesions
Source: Arthritis Res Ther. 2020 Apr 7;22:72. doi: 10.1186/s13075-020-02171-6 (PMC7137303; doi:10.1186/s13075-020-02171-6)
Supplement: Supplementary file 1 — Additional file 1. Supplementary tables. [file 13075_2020_2171_MOESM1_ESM.docx]

Table S1. Definition of signs and symptoms of GCA in vessels of interest

| Signs and symptoms of cranial lesions |
| --- |
| - Lesions in the temporal arteries and the maxillary artery.   Clinical manifestations include temporal artery tenderness, decreased pulse, hyperplasia, scalp tenderness, and headaches. Clinical manifestations include jaw claudication, mandibular pain, and glossodynia.   - Lesions in the ophthalmic artery.   Includes transiently reduced visual acuity, permanently reduced visual acuity, blindness, diplopia, visual field defects, other symptoms associated with ischemic optic neuropathy, and abnormal fundus findings.   - Lesions in the internal carotid artery, cerebral arteries, or basilar artery. Signs and symptoms due to arteritis in the internal carotid artery, cerebral arteries, and the basilar artery. Does not include lesions in carotid arteries or cerebral embolism due to cardiac lesions. Does not include arteriosclerotic lesions. |
| Signs and symptoms of LVLs   - Lesions in carotid, vertebral arteries, or brachiocephalic artery.   Signs and symptoms due to arterial stenosis, an aneurysm, or arterial dissection. Clinical manifestations include neck pain due to vasculitis, a bruit due to stenosis, ischemic symptoms due to stenosis (e.g., pain during neck movement, fatigability, and syncope), dizziness due to hyperactive carotid sinus reflex syndrome, syncope, and cerebral embolism related to thrombus formation as a result of arteritis.   - Lesions in a subclavian artery, axillary artery, or brachial artery.   Symptoms due to arterial stenosis, an aneurysm, or arterial dissection. Clinical manifestations include pain due to vasculitis, a bruit due to stenosis, decreased or absent radial or brachial pulse, blood pressure asymmetry >10 mm Hg, and ischemic symptoms due to stenosis (e.g., arm claudication). Subclavian steal syndrome due to a subclavian artery obstruction.   - Lesions in the thoracic aorta.   Signs and symptoms due to arteritis of the ascending aorta, aortic arch, or descending aorta. Symptoms due to arterial stenosis, an aneurysm, or arterial dissection. Includes clinical manifestations associated with rupture of an aortic aneurysm, aortic dissection, or atypical coarctation of the aorta.   - Aortic insufficiency.   Signs and symptoms due to annuloaortic ectasia or aortic insufficiency caused by vasculitis of the aortic valve. Includes progression of aortic insufficiency and symptoms of heart failure.   - Lesions in coronary arteries.   Signs and symptoms due to stenosis of the origin of the coronary arteries and lesions in the ascending aorta or symptoms due to arteritis of the coronary arteries.   - Lesions in pulmonary arteries.   Signs and symptoms due to arteritis of the pulmonary arteries. Includes clinical manifestations associated with pulmonary hypertension.   - Lesions in the abdominal aorta.   Signs and symptoms due to arteritis of the abdominal aorta, arterial stenosis, an aneurysm, or arterial dissection. Includes clinical manifestations associated with rupture of an aortic aneurysm, aortic dissection, or atypical coarctation of the aorta.   - Lesions in renal arteries.   Signs and symptoms due to arteritis of the renal arteries, arterial stenosis, an aneurysm, or arterial dissection. Clinical manifestations include an abdominal bruit suggesting renal artery stenosis, renovascular hypertension, ischemic nephropathy with bilateral renal artery stenosis, and renal failure. Does not include symptoms that are presumably due to arteriosclerosis.   - Lesions in iliac arteries or the femoral artery.   Signs and symptoms due to arteritis of the iliac arteries or femoral artery, arterial stenosis, an aneurysm, or arterial dissection. Clinical manifestations include decreased or absent pulse in the lower extremities due to stenosis and claudication. Does not include symptoms that are presumably due to arteriosclerosis. |

Table S2. Demographic and clinical features of the patient cohort for analysis of relapse at baseline

| Characteristics | Analyzed  patients^a^  n=119 | Enrolled  patients^b^  n=139 |
| --- | --- | --- |
| Age, years, mean ± SD, (n=119^c^) | 73.0 ± 7.7 | 73.8 ± 7.7 |
| Female patients, %, (n=119) | 67.2 | 66.9 |
| Weight, kg, mean ± SD, (n=119) | 51.4 ± 10.6 | 50.9 ± 10.4 |
| GCA ACR classification criteria, %, (n=119) | 76.5 | 78.4 |
| Modified GCA classification criteria, %, (n=119) | 99.2 | 99.3 |
| TAB performed, %, (n=119) | 58.8 | 61.9 |
| TAB positive, %, (n=119) | 49.6 | 50.4 |
| Imaging positive, %, (n=119) | 57.1 | 52.5 |
| Signs and symptoms of cranial lesions, %, (n=119) | 77.3 | 77.7 |
| Headache, %, (n=119) | 59.7 | 61.2 |
| Abnormal temporal artery, %, (n=119) | 59.7 | 59.0 |
| Jaw claudication, %, (n=119) | 33.6 | 36.0 |
| Visual disturbance, %, (n=119) | 23.5 | 23.7 |
| Visual loss, %, (n=119) | 2.5. | 4.3 |
| Signs and symptoms^d^ of LVL, %, (n=119) | 23.5 | 25.9 |
| Neck, %, (n=116) | 9.5 | 10.3 |
| Upper limbs, %, (n=116) | 10.3 | 11.8 |
| Lower limbs, %, (n=115) | 3.5 | 3.0 |
| Chest or abdominal bruit, %, (n=112) | 10.7 | 9.2 |
| Fever, %, (n=117) | 32.5 | 32.1 |
| Constitutional symptoms, %, (n=113) | 77.9 | 75.8 |
| Polymyalgia rheumatica, %, (n=119) | 42.0 | 41.7 |
| CRP, mg/dl, median (interquartile range), (n=119) | 6.9 (3.3-11.2) | 7.2 (3.3-11.2) |
| Ischemic heart disease, %, (n=118) | 5.9 | 7.2 |
| Cerebrovascular disease, %, (n=116) | 13.8 | 14.1 |
| Chronic lung disease, %, (n=118) | 6.8 | 8.0 |
| Hypertension, %, (n=119) | 42.9 | 44.9 |
| Diabetes Mellitus, %, (n=118) | 18.6 | 21.0 |
| Hyperlipidemia, %, (n=119) | 26.9 | 25.3 |
| Osteoporosis, %, (n=102) | 19.6 | 21.5 |

^a^Newly-diagnosed patients who were observed for 24 weeks or longer.

^b^Number of analyzed patients in each item.

^c^Enrolled newly-diagnosed GCA patients treated with glucocorticoids between 2007 and 2014 in a retrospective, multi-center registry. Number of patients using in the analysis of each item was shown in Table 1 in the manuscript.

^d^Signs and symptoms of neck included tenderness of carotid arteries, carotid bruit or neck claudication. Signs and symptoms of upper limb included arm claudication, decreased or absent radial pulse, or blood pressure asymmetry >10 mm Hg. Lower limb included leg claudication or decreased or absent pulse of lower limb.

LVLs, large-vessel lesions; LV, large-vessel; ACR, American College of Rheumatology; CRP, C-reactive protein.

Table S3. Associated factors with relapse in patients (n=106^*^) who achieved clinical remission for by week 24

|  | Univariable analysis | | Multivariable analysis | | |
| --- | --- | --- | --- | --- | --- |
|  | HR (95% CI) | p | HR (95% CI) | | p |
| Age, per one year increment | 1.02 (0.97-1.07) | 0.500 | 1.07 (1.00-1.13) | | 0.048 |
| Female | 1.16 (0.52-2.56) | 0.719 | 1.03 (0.46-2.32) | | 0.940 |
| Any cranial symptoms at baseline | 0.60 (0.27-1.37) | 0.228 | 0.92 (0.37-2.28) | | 0.850 |
| Polymyalgia rheumatica at baseline | 1.11 (0.52-2.34) | 0.793 | 1.17 (0.54-2.52) | | 0.688 |
| LVL at baseline | 2.92 (1.24-6.88) | 0.014 | 4.41 (1.62-12.0) | | 0.004 |
| Any lesions of aortic branches^b^ | 1.56 (0.74-3.27) | 0.240 |  |  | |
| Any lesions of aorta^c^ | 2.18 (1.04-4.58) | 0.04 |  |  | |
| Any structural vascular damage | 1.60 (0.70-3.62) | 0.265 |  |  | |
| Aneurysm of aorta | 2.34 (0.55-9.85) | 0.248 |  |  | |
| CRP at baseline per one mg/dl increment | 1.00 (0.94-1.07) | 0.911 |  |  | |
| Initial dose of PSL per  0.1 mg/kg/day increment | 1.15 (0.96-1.38) | 0.140 |  |  | |
| Immunosuppressive drug use at baseline | 1.51 (0.52-4.34) | 0.449 |  |  | |

^*^Patients who did not achieve clinical remission by week 24 were excluded from the analysis of relapse.

^b^Any lesions of aortic branches by imaging included lesions in carotid, vertebral, brachiocephalic, subclavian, axillary artery, pulmonary, renal, or iliac arteries.

^c^Any lesions of aorta by imaging included lesions in ascending aorta, aorta arch, descending thoracic aorta, or abdominal aorta.

^d^Any structural vascular damage included stenosis, dilatation or aneurysm in lesions of aortic branches and aorta.

LVL = large vessel lesions; CRP = C-reactive protein; PSL = prednisolone.

Table S4. Comparison of adverse events^*^ between the patients with and without LVL (n=119)

| Parameter | Patients with LVL  (n=68) | Patients without LVL  (n=51) |
| --- | --- | --- |
| Serious infections, n (%) | 9 (13.2%) | 10 (19.6%) |
| Bacterial pneumonia, n | 3 | 3 |
| Urinary tract infection, n | 1 | 2 |
| Sepsis, n | 0 | 1 |
| Pneumocystis pneumonia, n | 0 | 1 |
| Tuberculosis, n | 1 | 2 |
| Nontuberculous mycobacteriosis, n | 1 | 0 |
| Cytomegalovirus, n | 2 | 2 |
| Cryptococcal meningitis, n | 1 | 0 |
| Cardiac disorders, n (%) | 3 (4.4%) | 1 (1.9%) |
| Cerebrovascular events, n (%) | 2 (2.9%) | 0 |
| Bone fracture, n (%) | 3 (4.4%) | 2 (3.9%) |
| Gastrointestinal bleeding, n (%) | 2 (2.9%) | 0 |
| Exacerbation of diabetes mellitus, n (%) | 9 (13.2%) | 5 (9.8%) |
| Psychosis, n (%) | 4 (5.9%) | 2 (3.9%) |
| Glaucoma or a cataract, n (%) | 2 (2.9%) | 1 (2.0%) |

^*^Information about adverse events were collected for 0-52 weeks after start of treatment.
